# Recent advances in phototherapy for psoriasis

**DOI:** 10.12688/f1000research.8846.1

**Published:** 2016-07-13

**Authors:** Mio Nakamura, Benjamin Farahnik, Tina Bhutani

**Affiliations:** 1 Department of Dermatology, University of California San Francisco Psoriasis and Skin Treatment Center, San Francisco, California, USA; 2 University of Vermont College of Medicine, Burlington, Vermont, USA

**Keywords:** Psoriasis, Phototherapy, ultraviolet, UV

## Abstract

Phototherapy involves repeated exposure of the skin to ultraviolet light to treat various inflammatory skin conditions such as psoriasis. Recent studies have identified specific immunologic effects of phototherapy that may underlie phototherapy efficacy. Furthermore, recent advancements have been made in developing safe and effective targeted phototherapy modalities for difficult-to-treat areas such as scalp psoriasis. Targeted phototherapy in the form of the excimer laser holds potential for more aggressive, effective treatment and long-lasting remission of psoriasis. Phototherapy is now also used successfully with biologic agents as combination therapy to treat recalcitrant psoriasis. Therefore, though one of the oldest therapeutic modalities for psoriasis, phototherapy remains a mainstay treatment with promise for further advancement.

## Introduction

Phototherapy involves repeated exposure of the skin to ultraviolet (UV) light to treat various inflammatory skin conditions such as psoriasis, eczema, and vitiligo. This therapy is one of the oldest treatment modalities in dermatology, dating back to the ancient Egyptians, who used natural light in combination with herbal extracts to treat skin disease ^1^. Phototherapy continues to be a highly preferred treatment by dermatologists ^2^; however, its use has been decreasing in the past decades ^3^. The cause of this decline is likely multifactorial, including lack of training ^4^, low insurance reimbursement rates, and increase in cost of phototherapy treatments ^3^. Developments have been made in the past several decades to make phototherapy an effective, convenient, and accessible treatment option for patients; however, despite research efforts, there is still much to be learned about phototherapy, including mechanism of action and how best to maximize effectiveness of treatments. This review will focus on recent advances in phototherapy for the treatment of psoriasis, including new information on the possible impact of UV light on the immunologic environment, new methods of delivering targeted light treatments by efficient yet safe methods, and using phototherapy in combination with biologic agents to treat recalcitrant psoriasis.

There are three main types of phototherapy used for the treatment of psoriasis: broadband ultraviolet B (BB-UVB), narrowband ultraviolet B (NB-UVB), and psoralen plus ultraviolet A (PUVA). BB-UVB was the first to be developed, in the 1940s, and emits wavelengths of light of between 290 and 313 nm. NB-UVB, which emits wavelengths of between 308 and 313 nm, was developed in the late 1980s when studies demonstrated that wavelengths of around 311 nm were more effective than broad-spectrum UVB in clearing psoriasis. UVA, with wavelengths of 320 to 400 nm, is used in combination with psoralen, a photosensitizing medication, which can be taken orally prior to UVA exposure in systemic PUVA or applied topically in soak PUVA ^5^. NB-UVB is the most commonly used phototherapy modality today as it has a wider application across various dermatologic conditions, is easier to use, and has fewer side effects when compared with BB-UVB or PUVA ^6^.

Phototherapy is most commonly administered in the office setting in stand-up light booths. The starting dose of UV light is determined by the individual patient’s minimal erythema dose (MED) or by Fitzpatrick skin type, and subsequent dosing is increased as tolerated. Approximately 60% to 75% of patients with moderate to severe psoriasis achieve at least 75% improvement in the Psoriasis Area and Severity Index (PASI-75) ^7^. The various modes of phototherapy, including BB-UVB, NB-UVB, systemic PUVA, and soak PUVA, appear to have comparable efficacy ^8^. Phototherapy is relatively well tolerated, and the most common side effect is burning or itching. There is no overwhelming evidence for increased risk of skin cancers except in Caucasian patients who have undergone more than 250 treatments of systemic PUVA ^9^.

Phototherapy treatments in a hospital or office setting are inconvenient and unfeasible for some patients. Many patients have difficulty committing to the phototherapy regimen in the office because of time restraints, transportation problems, mobility issues, and high insurance co-pays ^10^. For these patients, home UV units have been developed. The use of home phototherapy for patients with psoriasis was first reported in 1979 in a study from Sweden in patients who lived in remote areas far from their nearest hospital phototherapy unit ^11^. Home phototherapy is associated with lower burden of treatment and increased patient satisfaction ^12^.

## Recent advances

### The immunoregulatory effects of phototherapy: possible pathways

Psoriasis is caused by abnormal interactions among innate immune cells, T cells, and keratinocytes, leading to activation of the T helper cell type 1/T helper cell type 17 (Th1/Th17) immune axes and related cytokines. This contributes to the hyperproliferation and inflammation seen in psoriasis. There are various mechanisms by which phototherapy may be effective for psoriasis ^13^. First, UV light induces apoptosis of keratinocytes and T cells in the epidermis and dermis ^14^. Second, UV light promotes immunosuppression by promoting migration of Langerhans cells out of the epidermis ^15^ as well as decreasing mast cell degranulation and histamine release ^16^. Lastly, UV light induces alterations in the cytokine profile of psoriasis.

Research in the last 5 years has led to a better understanding of the specific pathways and cytokines that are altered by phototherapy. Phototherapy shifts the immune response away from the Th1/Th17 pathway, toward the counter-regulatory Th2 axis ^17^. The Th1/Th17 pathway is suppressed by NB-UVB, leading to decreased levels of interleukin-12 (IL-12), IL-17, IL-20, IL-22, and IL-23 ^18^. These effects on cytokines appear to be systemic and not just localized to psoriatic lesions. PUVA and NB-UVB lower plasma levels of tumor necrosis factor-alpha, IL-17, IL-22, and IL-23 at the end of 6 weeks of treatment ^19^. Furthermore, regulatory T (Treg) cells of patients with severe psoriasis display an enhanced propensity to convert into IL-17A-producing cells, which is linked to loss of forkhead box P3 (Foxp3) ^20^. UVB increases Foxp3-positive Treg cells in psoriatic skin lesions ^21^. This increase in Foxp3 expression improves Treg cell stability and reduces pro-inflammatory Th1/Th17 cytokines in psoriatic skin lesions.

### Handheld phototherapy: targeting difficult-to-treat psoriasis in the office and at home

Whole-body phototherapy is limited in that there can be needless exposure of uninvolved skin and no benefit to unexposed skin (such as the hair-covered scalp). In the last decade, various portable and lightweight handheld phototherapy units have become available for the treatment of localized psoriasis in the office and at home ( Table 1) ^22, 23^. These handheld devices, as compared with full-body irradiance in a booth or by a panel, have the added benefit of limiting skin exposure to UV light. The handheld devices are useful for the treatment of scalp psoriasis as well as recalcitrant localized psoriasis plaques. For example, the Dermalight 90 by National Biological Corporation has a comb attachment that permits direct application of light to scalp lesions. Although large-scale clinical trials are lacking, such light combs appear to be efficacious with longer remission compared with topical treatments ^24, 25^. The handheld devices typically deliver NB-UVB, but some devices use BB-UVB or UVA. The DuaLight by Theralight, Inc. and Psoria-Light by Psoria-Shield can deliver both UVB and UVA, but these devices are for use only in an office setting.

**Table 1.** Handheld phototherapy devices ^11, 19^.

Series	Source	Treatment area, inches	Size, inches	Features
Dermalight 90 (National Biological Corporation)	NB-UVB (three lamps)	1.75 × 5	3.2 × 12.8 × 1.2	• Comb attachment for scalp treatment • Timer to monitor treatment length • Alert when energy from lamps gets low
Dermalume 2× (National Biological Corporation)	NB-UVB (two lamps)	3.3 × 5.4	4.1 × 7.5 × 2.8 (folded)	• Key lock • Timer that automatically turns lamps on/off • Emergency on/off switch
DermaPal (Daavlin)	NB-UVB UVA	8.25 × 1.75	2.75 × 9 × 5	• High-output lamps shorten treatment times • Highly accurate, integrated timer
DuaLight (Theralight) ^a^	NB-UVB BB-UVB UVA	0.75 × 0.75	6.5 × 16.5 × 11.5	• Minimal erythema dose and minimal phototoxic dose phototest modes • Built-in timer and calibrating port • Square aperture handpiece provides unobstructed view • Flexible liquid light guide provided • Disposable tip for hygienic single-patient use prevents cross-contamination
Levia (Daavlin)	UVB	0.67 × 0.67	8 × 6 × 11.5	• Touch-screen control panel • Built-in calibration port • LiteSpot and LiteBrush attachments • Light administered in small squares that can be tiled to treat the entire surface
Psoria-Light (Psoria- Shield) ^a^	NB-UVB, UVA	(0.45 square inches)	18 × 22 × 8.5	• LED (light emitting diode) source • Fast switching, on/off ability, not requiring a warm-up or cool-down period • High-definition digital camera to capture before and after photos of treatment sites • Touch technology to detect the patient’s treatment site before enabling UV dosage administration
100 Series Handheld Phototherapy (SolarC Systems)	NB-UVB BB-UVB (two lamps)	2.5 × 5	3.5 × 7 × 2.25	• Includes a carrying case • Includes aperture plates for precise localized treatment • Positioning arm and UV brush • Acrylic panel covering lamps • Timer with maximum 20 minutes • Switch lock

^a^Office use only. BB-UVB, broadband ultraviolet B; NB-UVB, narrowband ultraviolet B; UV, ultraviolet; UVA, ultraviolet A; UVB, ultraviolet B.

Two concerns for home administration of phototherapy are patient adherence and the possibility of overuse, underuse, and inappropriate use ^26^. Fortunately, various features have been developed in recent years to increase safety of home phototherapy. Features such as an integrated timer and automated calibration can limit the length of time of treatment sessions and number of treatment sessions between follow-up visits with the physician. However, proper screening of candidates and patient education continue to be important to ensure safety and compliance ^27^. For the physician, an increasing number of resources are now available to increase knowledge and ease of prescribing home phototherapy units ^23^.

### The excimer laser: a potential new indication and a novel dosimetry protocol

In addition to targeted therapy in the form of handheld devices, the 308 nm excimer laser was developed in 1997 as a targeted NB-UVB source for the treatment of psoriasis ^28^. The advantage to using the excimer laser is that because psoriasis plaques can take higher doses of light compared with normal skin, targeted treatment of psoriasis lesions using higher doses permits quicker time to clearance. In a multicenter open-label trial, 72% of patients with mild to moderate psoriasis achieved at least 75% improvement of the target plaque in an average of 6.2 treatments ^29^. Compared with traditional whole-body phototherapy, the excimer laser required fewer visits ^29^. In another study, 13 out of 26 patients with plaque-type psoriasis had continued clearance or long-term improvement after 1 year ^30^.

These initial studies tested the excimer laser in patients with localized psoriasis and thus the excimer laser is currently US Food and Drug Administration indicated for the treatment of mild to moderate psoriasis. However, the excimer laser may also be safe and effective for use in generalized, moderate to severe psoriasis ^31^. In a single-center pilot study, patients with greater than 10% but less than 30% body surface area involvement (moderate to severe psoriasis) were treated with excimer laser twice weekly for 12 weeks for a total of 24 treatment sessions. Fifty-four percent achieved PASI-75 and 83% achieved PASI-50, which was maintained without further treatment for 6 months ^32^. However, the downside to treating moderate to severe psoriasis is the long duration required per session to treat a large body surface area, which may not be feasible in many dermatology office settings.

Another area of recent advancement for the excimer laser is in the dosimetry protocol. The current accepted starting dose is typically based on the patient’s MED or Fitzpatrick skin type as well as location and thickness of the psoriatic lesion ^28^. The dose is increased as tolerated according to degree of erythema at each treatment session ^28^. A limitation to the current dosing method is that because the MED and Fitzpatrick skin type are based on normal skin, the starting dose is typically an underestimate. It typically takes 5 to 10 treatments of incremental dose increase just to reach the ideal maximum dose of the plaque. A recent study has attempted to develop a new dosing protocol called “plaque-based sub-blistering dosimetry”, in which the patient’s psoriatic plaque, rather than uninvolved skin, is tested with incrementally increasing doses to determine the dose at which blistering is observed ^32^. The patients then are treated just below that dose. This allows plaques to be treated at the most aggressive possible dose (though still a tolerable one) to achieve faster clearing. The starting dose used in this protocol is 8 to 16 times the MED, compared with 2 to 4 times the MED of the currently accepted dosing protocol. The use of this “plaque-based sub-blistering dosimetry” can result in achievement of PASI-75 in a patient with moderate psoriasis after only two treatment sessions ^32^.

### Phototherapy and biologic agents: combination therapy for recalcitrant psoriasis

Although several biologic agents showing excellent efficacy in the treatment of moderate to severe psoriasis have been developed in the last decade, phototherapy appears to play an important role in a subset of patients with severe, recalcitrant psoriasis despite treatment with a biologic agent. Several studies have demonstrated the efficacy of using etanercept and NB-UVB in combination ^33– 38^. These studies evaluated this combination therapy in patients who had not previously received treatment, patients who had an inadequate response with etanercept alone (50 mg once-weekly or 50 mg twice-weekly dosing), or patients who had an inadequate response to NB-UVB alone. Overall, combination therapy was superior and time to clearance was reduced. A study by Lynde *et al*. also demonstrated the importance of high adherence to the NB-UVB regimen in order to achieve significant clinical improvement ^36^. High adherence to the NB-UVB regimen was defined as missing not more than two treatments in any 4-week period.

One study to date has failed to establish efficacy of combination therapy of etanercept and NB-UVB. This head-to-head pilot study by Park *et al*., who examined combination treatment with NB-UVB and etanercept 50 mg twice weekly compared with etanercept monotherapy, did not demonstrate significantly enhanced improvement with combination therapy ^39^. However, this study was limited by a small sample size of 13 patients. Furthermore, the patients all had a body mass index (BMI) of greater than 30, and studies have reported a suboptimal response to etanercept in psoriasis patients with a BMI of greater than 30 ^40, 41^.

Adalimumab or ustekinumab in combination with NB-UVB has also been investigated in a limited number of studies. A study by Bagel *et al*. evaluated the combination of adalimumab and NB-UVB ^42^, and another study, by Wolf *et al*., evaluated adalimumab and the excimer laser ^43^. Both studies demonstrated that phototherapy significantly accelerates therapeutic response and improves the clearance of psoriatic lesions in patients who received adalimumab treatment. One study to date has evaluated the combination of ustekinumab with the excimer laser ^44^. This was an intra-individual, half-body comparison study in which PASI-75 was achieved significantly more often on the UV-irradiated half than on the non-irradiated half at week 6 in patients on ustekinumab.

In general, combination therapy involving a biologic agent with NB-UVB phototherapy was very well tolerated. The most common side effect was erythema. Although long-term studies do not currently exist, no skin cancers were reported throughout the duration of the above-mentioned trials.

## Conclusions

Although phototherapy is one of the oldest therapeutic modalities for psoriasis, it remains a mainstay treatment that holds promise for further advancement. Recent studies have delineated specific immunologic mediators that are affected by UV light. Such findings may lead to identification of targets for future psoriasis therapies.

Another area of recent advancement is in targeted phototherapy. Targeted phototherapy is advantageous in that it is a user-friendly tool that can irradiate difficult-to-treat areas as well as deliver higher doses of UV light compared with the traditional, whole-body phototherapy treatments. Currently, there are limited studies evaluating efficacy of handheld UVB use, and future studies should be conducted to evaluate long-term efficacy, cost effectiveness, and patient satisfaction. Interesting topics of future studies include the development of an effective dosimetry protocol for handheld UV devices that can be used safely both in the office setting and by patients at home.

Future areas of research for the excimer laser include large-scale, long-term studies evaluating its use for the treatment of moderate to severe psoriasis as a potential new indication. The evaluation of safety, efficacy, and practicality of the “plaque-based sub-blistering dosimetry” in a large-scale, long-term study is also of interest.

Lastly, NB-UVB appears to be a safe and effective treatment to be used concomitantly with biologic agents for recalcitrant psoriasis. Long-term, large-scale studies are needed to better understand safety of the use of this combination therapy, especially pertaining to the risk of non-melanoma skin cancer. Similar studies using BB-UVB and PUVA are lacking and are of interest for future research. Cost effectiveness, patient satisfaction, and quality of life with this combination therapy should also be studied.

## Abbreviations

BB-UVB, broadband ultraviolet B; BMI, body mass index; Foxp3, forkhead box P3; IL, interleukin; MED, minimal erythema dose; NB-UVB, narrowband ultraviolet B; PASI, Psoriasis Area and Severity Index; PUVA, psoralen plus ultraviolet A; Th1, T helper cell type 1; Th17, T helper cell type 17; Treg, regulatory T; UV, ultraviolet; UVA, ultraviolet A; UVB, ultraviolet B.
